# 2-Carbamylpyridinium tetra­chlorido­ferrate(III)

**DOI:** 10.1107/S1600536809040148

**Published:** 2009-10-17

**Authors:** Anne Nielsen, Christine J. McKenzie, Andrew D. Bond

**Affiliations:** a University of Southern Denmark, Department of Physics and Chemistry, Campusvej 55, 5230 Odense M, Denmark

## Abstract

The title compound, (C_6_H_7_N_2_O)[FeCl_4_], contains two carbamylpyridinium (picolinamidinium) cations, which are linked into chains by N^+^—H⋯O hydrogen bonds formed between protonated pyridyl N atoms and carbonyl groups. Tetra­chloridoferrate(III) anions lie between these chains, accepting N—H⋯Cl hydrogen bonds from both H atoms of the picolinamidium –NH_2_ group.

## Related literature

For related structures containing picolinamidium cations, see: Uçar *et al.* (2004 ▶); Gotoh *et al.* (2009 ▶).
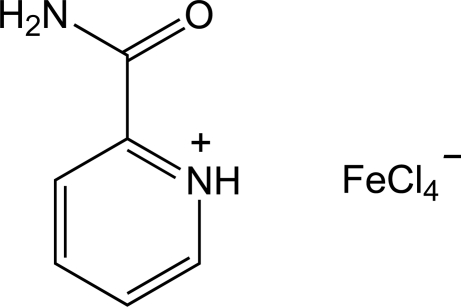


## Experimental

### 

#### Crystal data

(C_6_H_7_N_2_O)[FeCl_4_]
*M* *_r_* = 320.79
Monoclinic, 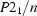

*a* = 13.5252 (8) Å
*b* = 6.1704 (3) Å
*c* = 14.1165 (7) Å
β = 93.853 (2)°
*V* = 1175.44 (11) Å^3^
*Z* = 4
Mo *K*α radiation
μ = 2.16 mm^−1^
*T* = 180 K
0.40 × 0.30 × 0.20 mm

#### Data collection

Bruker APEXII CCD diffractometer
Absorption correction: multi-scan (*SADABS*; Sheldrick, 2003 ▶) *T*_min_ = 0.484, *T*_max_ = 0.672
15093 measured reflections
2808 independent reflections
2420 reflections with *I* > 2σ(*I*)
*R*_int_ = 0.024

#### Refinement

*R*[*F* ^2^ > 2σ(*F* ^2^)] = 0.020
*wR*(*F* ^2^) = 0.049
*S* = 1.05
2808 reflections
140 parameters
2 restraints
H atoms treated by a mixture of independent and constrained refinement
Δρ_max_ = 0.26 e Å^−3^
Δρ_min_ = −0.23 e Å^−3^

### 

Data collection: *APEX2* (Bruker, 2004 ▶); cell refinement: *SAINT* (Bruker, 2003 ▶); data reduction: *SAINT*; program(s) used to solve structure: *SHELXTL* (Sheldrick, 2008 ▶); program(s) used to refine structure: *SHELXTL*; molecular graphics: *SHELXTL*; software used to prepare material for publication: *SHELXTL*.

## Supplementary Material

Crystal structure: contains datablocks global, I. DOI: 10.1107/S1600536809040148/zq2012sup1.cif

Structure factors: contains datablocks I. DOI: 10.1107/S1600536809040148/zq2012Isup2.hkl

Additional supplementary materials:  crystallographic information; 3D view; checkCIF report

## Figures and Tables

**Table 1:** Hydrogen-bond geometry (Å, °)

*D*—H⋯*A*	*D*—H	H⋯*A*	*D*⋯*A*	*D*—H⋯*A*
N1—H1⋯O1^i^	0.85 (2)	2.00 (2)	2.7234 (16)	142 (2)
N2—H22⋯Cl3	0.84 (1)	2.78 (2)	3.5710 (15)	160 (2)
N2—H21⋯Cl1^ii^	0.82 (1)	2.69 (2)	3.4811 (14)	163 (2)

Symmetry codes: (i) 
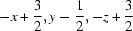
; (ii) 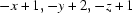.

**Fig. 1.**
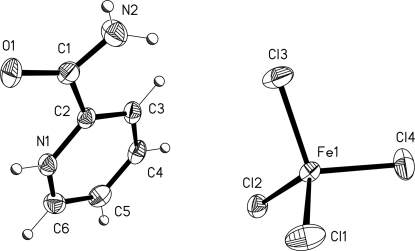
Molecular structure with displacement ellipsoids shown at 50% probability for non-H atoms.

**Fig. 2.**
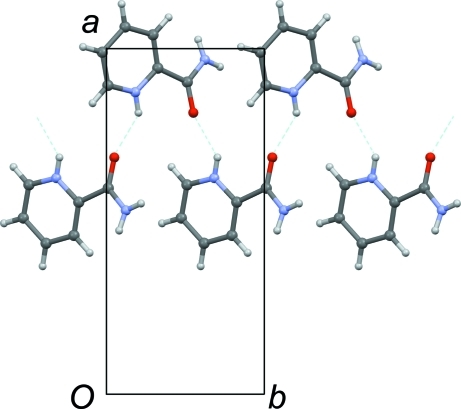
Picolinamidium cations linked into chains along the b axis by N+—H···O hydrogen bonds.

**Fig. 3.**
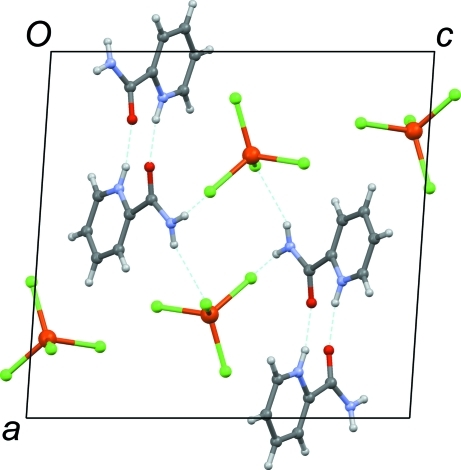
Unit-cell contents projected along the b axis, showing FeCl4- anions between the hydrogen-bonded chains of picolinamidium cations.

## supplementary crystallographic information

### Comment

Picolinamidium cations are present in two other structures in the Cambridge Structural Database. One structure (refcode: EYIXAL; Uçar *et al.*, 2004) includes squarate anions, C_4_HO_4_^-^, and contains planar picolinadinium:squarate layers in which all three N—H donors in picolinadinium form hydrogen bonds to squarate. The other (refcode: POVZEG; Gotoh *et al.*, 2009) contains chloranilate anions, C_6_HCl_2_O_4_^-^, in which all three N—H donors in picolinadinium form hydrogen bonds to chloranilate.

### Experimental

Picolinamide (35 mg, 28 mmol) was dissolved in acetonitrile (2.75 ml) and anhydrous FeCl_3_ (46 mg, 28 mmol) trimethylamine-*N*-oxide (32 mg, 28 mmol) and concentrated hydrochloric acid (0.2 ml) were added. After one week, a few milligrams of the title compound were deposited as yellow crystals.

### Refinement

H atoms bound to C atoms were placed in idealized positions with C—H = 0.95 Å and refined as riding with *U*_iso_(H) = 1.2*U*_eq_(C). H atoms bound to N were located in difference Fourier maps and refined with isotropic displacement parameters. The distances N2—H21 and N2—H22 were restrained to a common refined value, with an s.u. of 0.01 Å, and atom H1 was refined without restraint.

### Crystal data

**Table:**

(C_6_H_7_N_2_O)[FeCl_4_]	*F*(000) = 636
*M**_r_* = 320.79	*D*_x_ = 1.813 Mg m^−^^3^
Monoclinic, *P*2_1_/*n*	Mo *K*α radiation, λ = 0.71073 Å
Hall symbol: -P 2yn	Cell parameters from 8949 reflections
*a* = 13.5252 (8) Å	θ = 2.2–28.1°
*b* = 6.1704 (3) Å	µ = 2.16 mm^−^^1^
*c* = 14.1165 (7) Å	*T* = 180 K
β = 93.853 (2)°	Block, yellow
*V* = 1175.44 (11) Å^3^	0.40 × 0.30 × 0.20 mm
*Z* = 4	

### Data collection

**Table:**

Bruker–Nonius X8 APEXII CCD diffractometer	2808 independent reflections
Radiation source: fine-focus sealed tube	2420 reflections with *I* > 2σ(*I*)
graphite	*R*_int_ = 0.024
Thin–slice ω and φ scans	θ_max_ = 28.3°, θ_min_ = 3.6°
Absorption correction: multi-scan (*SADABS*; Sheldrick, 2003)	*h* = −17→15
*T*_min_ = 0.484, *T*_max_ = 0.672	*k* = −7→8
15093 measured reflections	*l* = −16→18

### Refinement

**Table:**

Refinement on *F*^2^	Primary atom site location: structure-invariant direct methods
Least-squares matrix: full	Secondary atom site location: difference Fourier map
*R*[*F*^2^ > 2σ(*F*^2^)] = 0.020	Hydrogen site location: inferred from neighbouring sites
*wR*(*F*^2^) = 0.049	H atoms treated by a mixture of independent and constrained refinement
*S* = 1.05	*w* = 1/[σ^2^(*F*_o_^2^) + (0.0236*P*)^2^ + 0.2718*P*] where *P* = (*F*_o_^2^ + 2*F*_c_^2^)/3
2808 reflections	(Δ/σ)_max_ = 0.002
140 parameters	Δρ_max_ = 0.26 e Å^−^^3^
2 restraints	Δρ_min_ = −0.23 e Å^−^^3^

### Special details

**Table:**

Geometry. All e.s.d.'s (except the e.s.d. in the dihedral angle between two l.s. planes) are estimated using the full covariance matrix. The cell e.s.d.'s are taken into account individually in the estimation of e.s.d.'s in distances, angles and torsion angles; correlations between e.s.d.'s in cell parameters are only used when they are defined by crystal symmetry. An approximate (isotropic) treatment of cell e.s.d.'s is used for estimating e.s.d.'s involving l.s. planes.
Refinement. Refinement of *F*^2^ against ALL reflections. The weighted *R*-factor *wR* and goodness of fit *S* are based on *F*^2^, conventional *R*-factors *R* are based on *F*, with *F* set to zero for negative *F*^2^. The threshold expression of *F*^2^ > σ(*F*^2^) is used only for calculating *R*-factors(gt) *etc*. and is not relevant to the choice of reflections for refinement. *R*-factors based on *F*^2^ are statistically about twice as large as those based on *F*, and *R*- factors based on ALL data will be even larger.

### Fractional atomic coordinates and isotropic or equivalent isotropic displacement parameters (Å^2^)

**Table:**

	*x*	*y*	*z*	*U*_iso_*/*U*_eq_	
O1	0.68522 (7)	1.05039 (17)	0.72326 (8)	0.0328 (2)	
N1	0.62577 (9)	0.68301 (19)	0.79936 (8)	0.0223 (2)	
H1	0.6851 (13)	0.703 (3)	0.7851 (11)	0.029 (4)*	
N2	0.53208 (10)	1.1233 (2)	0.65734 (10)	0.0332 (3)	
H21	0.5522 (13)	1.219 (3)	0.6233 (12)	0.044 (5)*	
H22	0.4739 (11)	1.081 (3)	0.6457 (13)	0.045 (5)*	
C1	0.59653 (10)	1.0119 (2)	0.71214 (10)	0.0236 (3)	
C2	0.55609 (10)	0.8255 (2)	0.76671 (9)	0.0201 (3)	
C3	0.45980 (10)	0.7950 (2)	0.78930 (9)	0.0229 (3)	
H3A	0.4095	0.8939	0.7674	0.027*	
C4	0.43698 (10)	0.6185 (2)	0.84436 (10)	0.0251 (3)	
H4A	0.3707	0.5966	0.8608	0.030*	
C5	0.51019 (11)	0.4743 (2)	0.87552 (10)	0.0279 (3)	
H5A	0.4948	0.3523	0.9128	0.034*	
C6	0.60612 (11)	0.5101 (2)	0.85165 (10)	0.0266 (3)	
H6A	0.6576	0.4128	0.8722	0.032*	
Fe1	0.283137 (14)	0.53471 (3)	0.540664 (13)	0.02141 (7)	
Cl1	0.38209 (3)	0.39866 (6)	0.43917 (3)	0.04193 (11)	
Cl2	0.30476 (3)	0.37620 (6)	0.67908 (2)	0.02817 (9)	
Cl3	0.31100 (3)	0.88308 (6)	0.55199 (3)	0.03815 (10)	
Cl4	0.12782 (3)	0.49004 (7)	0.48904 (3)	0.03714 (10)	

### Atomic displacement parameters (Å^2^)

**Table:**

	*U*^11^	*U*^22^	*U*^33^	*U*^12^	*U*^13^	*U*^23^
O1	0.0187 (5)	0.0355 (6)	0.0441 (6)	−0.0075 (4)	0.0024 (5)	0.0018 (5)
N1	0.0161 (6)	0.0268 (6)	0.0242 (6)	−0.0002 (5)	0.0030 (5)	−0.0048 (5)
N2	0.0235 (7)	0.0325 (7)	0.0440 (8)	−0.0003 (6)	0.0041 (6)	0.0106 (6)
C1	0.0208 (7)	0.0231 (7)	0.0276 (7)	−0.0014 (5)	0.0058 (6)	−0.0055 (6)
C2	0.0186 (6)	0.0224 (6)	0.0194 (6)	−0.0013 (5)	0.0007 (5)	−0.0066 (5)
C3	0.0181 (7)	0.0290 (7)	0.0212 (7)	−0.0001 (5)	−0.0002 (5)	−0.0062 (6)
C4	0.0195 (7)	0.0341 (8)	0.0221 (7)	−0.0061 (6)	0.0048 (6)	−0.0083 (6)
C5	0.0337 (8)	0.0273 (7)	0.0232 (7)	−0.0047 (6)	0.0050 (6)	−0.0023 (6)
C6	0.0286 (8)	0.0259 (7)	0.0250 (7)	0.0032 (6)	−0.0001 (6)	−0.0015 (6)
Fe1	0.02269 (12)	0.01900 (10)	0.02251 (11)	−0.00276 (7)	0.00133 (8)	−0.00228 (8)
Cl1	0.0585 (3)	0.03076 (19)	0.0393 (2)	0.00053 (18)	0.0235 (2)	−0.00672 (16)
Cl2	0.03014 (19)	0.03054 (18)	0.02317 (18)	−0.00099 (14)	−0.00311 (14)	−0.00080 (14)
Cl3	0.0468 (2)	0.01959 (17)	0.0481 (2)	−0.00492 (15)	0.00358 (19)	−0.00486 (16)
Cl4	0.0283 (2)	0.0439 (2)	0.0376 (2)	−0.00929 (16)	−0.00973 (16)	0.01353 (17)

### Geometric parameters (Å, °)

**Table:**

O1—C1	1.2225 (17)	C3—H3A	0.950
N1—C6	1.3341 (19)	C4—C5	1.381 (2)
N1—C2	1.3473 (18)	C4—H4A	0.950
N1—H1	0.85 (2)	C5—C6	1.380 (2)
N2—C1	1.319 (2)	C5—H5A	0.950
N2—H21	0.82 (1)	C6—H6A	0.950
N2—H22	0.84 (1)	Fe1—Cl3	2.1863 (4)
C1—C2	1.5073 (19)	Fe1—Cl2	2.1870 (4)
C2—C3	1.3744 (18)	Fe1—Cl1	2.1923 (4)
C3—C4	1.385 (2)	Fe1—Cl4	2.1941 (4)
			
C6—N1—C2	123.39 (13)	C5—C4—C3	120.23 (13)
C6—N1—H1	118.4 (11)	C5—C4—H4A	119.9
C2—N1—H1	118.2 (11)	C3—C4—H4A	119.9
C1—N2—H21	119.2 (13)	C6—C5—C4	119.00 (13)
C1—N2—H22	121.9 (13)	C6—C5—H5A	120.5
H21—N2—H22	117.1 (19)	C4—C5—H5A	120.5
O1—C1—N2	125.32 (14)	N1—C6—C5	119.25 (14)
O1—C1—C2	117.99 (13)	N1—C6—H6A	120.4
N2—C1—C2	116.68 (12)	C5—C6—H6A	120.4
N1—C2—C3	118.90 (13)	Cl3—Fe1—Cl2	111.243 (17)
N1—C2—C1	113.78 (12)	Cl3—Fe1—Cl1	108.329 (17)
C3—C2—C1	127.24 (12)	Cl2—Fe1—Cl1	111.207 (17)
C2—C3—C4	119.22 (13)	Cl3—Fe1—Cl4	107.713 (18)
C2—C3—H3A	120.4	Cl2—Fe1—Cl4	107.946 (16)
C4—C3—H3A	120.4	Cl1—Fe1—Cl4	110.350 (19)
			
C6—N1—C2—C3	1.15 (19)	N1—C2—C3—C4	−0.38 (19)
C6—N1—C2—C1	178.00 (12)	C1—C2—C3—C4	−176.75 (13)
O1—C1—C2—N1	−19.00 (18)	C2—C3—C4—C5	−0.5 (2)
N2—C1—C2—N1	162.32 (12)	C3—C4—C5—C6	0.6 (2)
O1—C1—C2—C3	157.53 (14)	C2—N1—C6—C5	−1.0 (2)
N2—C1—C2—C3	−21.1 (2)	C4—C5—C6—N1	0.1 (2)

### Hydrogen-bond geometry (Å, °)

**Table:**

*D*—H···*A*	*D*—H	H···*A*	*D*···*A*	*D*—H···*A*
N1—H1···O1^i^	0.85 (2)	2.00 (2)	2.7234 (16)	142 (2)
N2—H22···Cl3	0.84 (1)	2.78 (2)	3.5710 (15)	160 (2)
N2—H21···Cl1^ii^	0.82 (1)	2.69 (2)	3.4811 (14)	163 (2)

Symmetry codes: (i) −*x*+3/2, *y*−1/2, −*z*+3/2; (ii) −*x*+1, −*y*+2, −*z*+1.
